# A Review of Behavioral Economic Manipulations Affecting Drug versus Nondrug Choice in Rats

**DOI:** 10.1007/s40614-025-00445-5

**Published:** 2025-04-07

**Authors:** David N. Kearns

**Affiliations:** Psychology Department, American University, 4400 Massachusetts Avenue NW, Washington, DC 20016 USA

**Keywords:** Behavioral economics, Behavioral pharmacology, Drug self-administration, Animal models, Choice, Demand

## Abstract

Many recent studies have investigated rats’ choice between drug and nondrug reinforcers to model variables influencing drug taking in humans. As research using this model accumulates, the complexity of factors affecting drug choice has become increasingly apparent. This review applies a behavioral economic perspective to research that has used this model. The focus is on experiments that have manipulated behavioral economic variables in studies of rats’ choice between drugs like cocaine or heroin and nondrug reinforcers like saccharin or social interaction. Price effects, reinforcer interactions (i.e., as substitutes or complements), economy type, and income effects are described. Results of experiments testing the impact of these variables on rats’ choice are presented and analyzed. Although rats’ behavior in this model often conforms well with behavioral economic principles, there have also been instances where further explanation is required. By appreciating the behavioral economic context in which rats’ choice between drug and nondrug reinforcers occurs, and by recognizing that both consequences and antecedents can play important roles in this behavior, our understanding of the complexity of factors involved in drug choice can be increased.

Many studies over the past 15 years have investigated choice between drug and nondrug reinforcers in rats (Ahmed, 2018; Banks & Negus, 2012, 2017; Townsend et al., 2021). This interest in choice has been stimulated by the recognition that animal models of drug taking where the drug is the only option may not best model the human situation, where nondrug alternatives compete with drugs for a share of the allocation of behavior (Ahmed, 2010; Banks & Negus, 2017; Townsend et al., 2021). Various drug reinforcers have been used, including psychostimulants (e.g., Cantin et al., 2010; Lenoir et al., 2007; Tunstall et al., 2014; Venniro et al., 2021), opioids (e.g., Lenoir et al., 2013; Schwartz et al., 2017; Townsend et al., 2019a, 2019b; Venniro et al., 2018), alcohol (e.g., Augier et al., 2018; Pelloux & Baunez, 2017; Russo et al., 2018), and nicotine (Huynh et al., 2017). An even greater variety of nondrug alternatives have been used, including food pellets (e.g., Bossert et al., 2022; Caprioli et al., 2017; Giannotti et al., 2022; Heinsbroeck et al., 2021; Kearns et al., 2017; Kerstetter et al., 2012; Perry et al., 2013; Tunstall & Kearns, 2015; Venniro et al., 2016), water (Vandaele et al., 2019), sucrose (e.g., Lenoir et al., 2007; Rossi et al., 2020; Vandaele & Ahmed, 2022; Vandaele et al., 2016, 2022), saccharin (e.g., Cantin et al., 2010; Kearns et al., 2017; Lenoir et al., 2007, 2013, 2023; Schwartz et al., 2017; Vandaele et al., 2016, 2021, 2022), diluted Ensure (e.g., Moerke et al., 2022; Thomsen et al., 2013, 2022; Townsend et al., 2019a, 2019b), social interaction (e.g., Chow et al., 2022; Marcus et al., 2022; Papastrat et al., 2024; Smith et al., 2023; St. Onge et al., 2022; Venniro et al., 2018, 2019, 2020, 2021), the opportunity to escape/avoid shock (Marcus & Banks, 2023; Marcus et al., 2024), and a period of timeout from an avoidance schedule (Beasley et al., 2024).

Although many studies have found that availability of a nondrug alternative reduces allocation of behavior to the drug and that most rats prefer the nondrug alternative (e.g., Cantin et al., 2010; Tunstall et al., 2014), this is not always the case (e.g., Augier et al., 2023; Beasley et al., 2024; Bird et al., 2024; Heinsbroeck et al., 2021; Marchant et al., 2023; Peters , 2022). Rats’ choice behavior depends on the particular combinations of drug and nondrug reinforcers offered as well as various features of both the immediate choice situation as well as the broader context within which choices are made. Below the surface of a seemingly straightforward task that is presented to the rat—choose between a drug and nondrug reinforcer—there can be a great deal of complexity (Kearns, 2023).

This review will apply a behavioral economic perspective to choice between drug and nondrug reinforcers in rats to make sense of some of this complexity. The term behavioral economics has been used in different ways. In one type of behavioral economics, psychological concepts are applied to explain the often irrational (from an economic standpoint) choices made by humans (e.g., Thaler, 2016; Tversky & Kahneman, 1981). In a second type, which has been called operant behavioral economics (Foxall, 2016), economic principles are used to help describe and explain the operant behavior of animals and humans (e.g., Allison, 1983; Hursh, 1980; Rachlin et al., 1976). Operant behavioral economics includes many subareas of focus, including the modeling of demand (e.g., Hursh & Silberberg, 2008), delay discounting (e.g., Ainslie, 1991; Green & Myerson, 1993), and choice (Herrnstein, 1990). The present review applies selected concepts from this second type of behavioral economics to the study of drug versus nondrug alternative choice in rats.

The focus is on rat studies because Banks and Negus (2012, 2017) have previously provided excellent and comprehensive reviews covering the nonhuman primate literature on drug versus nondrug alternative choice. Of the 40 preclinical drug versus nondrug alternative choice studies performed at the time of the 2012 Banks and Negus review, only seven used rats and all others used primates (see Table 1, Banks & Negus, 2012). The surge of drug versus nondrug choice studies using rats began around 2010 (e.g., see publication years of the rat studies cited in first two paragraphs above), which is the year that Ahmed published a seminal article advocating for the use of choice in drug self-administration studies (Ahmed, 2010). The present review is not intended to cover every possible variable that can affect choice between drug and nondrug reinforcers in rats, but instead illustrate with examples how application of several behavioral economic concepts can further understanding of the processes involved in drug choice. In particular, the role of price effects, substitute/complement interactions, economy type, and income effects will be explored. Experiments in rats that have manipulated these behavioral economic variables will be described and findings will be discussed. The behavioral economic concepts have been developed over many years by innovators in the field (see, for example, Allison, 1983; Bickel et al., 1995; Bickel et al., 2000; Hursh, 1980, 1984, 1991, 2014; Hursh & Silberberg, 2008; Green & Freed, 1993; Green & Rachlin, 1991; Kagel et al., 1975; Rachlin et al., 1976). It is hoped that the application of these behavioral economic principles to rat choice studies will provide some insight into the rat model of drug taking, and by extension, understanding of the processes that could also be responsible for drug choice in humans.

Before getting into the experiments, first a comment on reinforcer magnitude will be made. In the studies to be described in detail below, the doses of cocaine or heroin used were on the descending limb of the fixed ratio (FR) 1 self-administration dose–response curves generated by others (e.g., Carroll & Lac, 1997; Gancarz et al., 2012; Martin et al., 1996; Raleigh et al., 2014). More precisely quantifying the relative reinforcing values of different doses of different drugs or of different drugs versus nondrug reinforcers is difficult. In choosing doses or nondrug alternative reinforcer magnitudes for most of the studies to be described in detail below, the goal was not to equate the reinforcing strengths of the two reinforcers. Instead, the more modest goal of nonexclusive preference under baseline (e.g., equal prices) conditions typically determined reinforcer magnitude (including dose) decisions. The rationale for attempting to produce nonexclusive preference under baseline conditions is that, if the reinforcer magnitudes used were such that rats exclusively chose one option or the other, floor or ceiling effects could mask the potential effect of the main independent variable of interest (e.g., economy type, income). Nevertheless, it should be noted that because often only one dose or nondrug alternative reinforcer magnitude was used, it is unknown whether similar effects would be observed with different combinations. It might be predicted, on the basis of prior studies finding orderly effects of dose on choice (e.g., see reviews by Banks & Negus, 2012, 2017), how results might change if different doses were used. This cannot be known for certain, however, until tested in additional experiments.

## Price Effects in Drug versus Nondrug Alternative Choice Studies

**Fig. 1:**
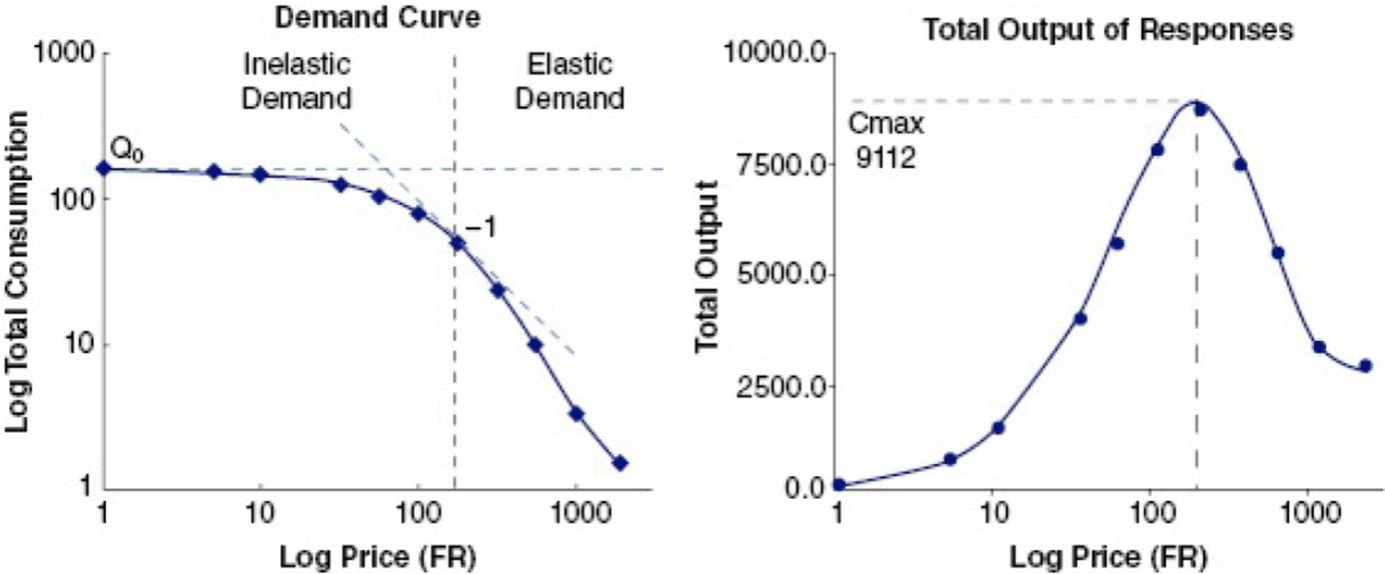
From Hursh (2014), the left panel shows a hypothetical demand curve representing consumption as a function of price and the right panel shows total response output as a function of price. The dashed vertical line in both panels represents P_max_. The left panel shows that demand elasticity shifts from inelastic to elastic at Pmax; the right panel shows that response output is maximal (O_max_) at P_max_. Reprinted from Hursh (2014) with permission from John Wiley & Sons

Each section will begin with a brief description of a behavioral economic concept. (For more in-depth discussion, the reader is referred to the sources noted above.) The first of these is price. Humans pay for things with money (usually), but rats pay for reinforcers with operant responses, typically lever presses. As the price of a good (a reinforcer) increases, consumption of that reinforcer typically decreases. This relation is described by the law of demand. Consumption can be plotted across a range of prices to produce a demand curve (see left panel of Fig. 1, reprinted from Hursh, 2014). At low prices, increases in price usually result in relatively small decreases in consumption. Demand is inelastic for this part of the curve. As prices increase, the subject makes more responses in an effort to defend consumption (see right panel of Fig. 1). A price is eventually reached where the subject no longer increases the number of responses it makes. This price is called P_max_. At prices beyond P_max_, further increases in price produce relatively large decreases in consumption. Demand is elastic for this part of the curve. Demand for goods that are thought of as luxuries becomes elastic sooner than demand for goods typically considered necessities. The rate at which demand becomes elastic with price increases varies for different reinforcers and can be precisely quantified by Hursh and Silberberg’s (2008) exponential demand model:

1 $$\text{log }Q=\text{log }{Q}_{0} + k\left({e}^{-aQ}{0}^{C}-1\right),$$

where *Q* is quantity consumed, *Q*_0_ is consumption as price approaches 0, *k* is a constant defining the consumption range in log units, *C* is cost, and* α* is rate of decrease in consumption. The Hursh and Silberberg model was used to fit the demand curves presented later in this article.

The foregoing describes behavior when considering responding for a single reinforcer, as in, for example, experiments where a rat is given only one lever that it can press to obtain a drug infusion with no other options. The effects of price are also at work in choice procedures where the rat can allocate behavior between a drug and nondrug alternative. The effects of price become more complicated in choice situations where the two reinforcers interact as substitutes or complements, as described in detail later. For now, the simpler case where the drug and nondrug alternatives do not interact, or where aspects of the procedure minimize potential interactions, will be considered.

In many drug versus nondrug alternative choice experiments in rats, a mutually exclusive discrete-trials procedure with relatively long (e.g., 8–10 min) intertrial intervals (ITIs) is commonly used (e.g., Cantin et al., 2010; Marchant et al., 2023; Venniro et al., 2018, 2019). Spacing of trials decreases (but may not eliminate) the potential influence of reinforcer interactions (Vandaele et al., 2016). Each trial begins with the insertion of two levers. Pressing one results in the drug; pressing the other results in presentation of the nondrug alternative. This choice opportunity represents one trial, which is followed by an ITI where neither lever is available. The next trial begins with insertion of the two levers again. At first, the price is typically one lever press (or sometimes two) per reinforcer. The number of reinforcers of each type that the rat obtains is often taken as an indication of its preference (e.g., see Cantin et al., 2010; Lenoir et al., 2007; Tunstall & Kearns, 2014, 2016; Venniro et al., 2018).

**Fig. 2:**
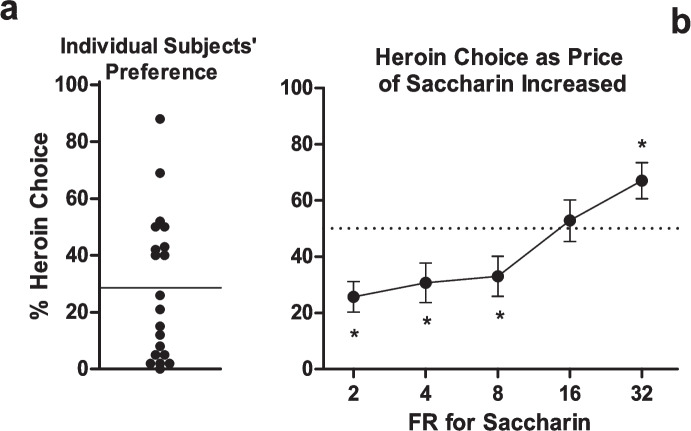
Results from rats choosing between heroin and saccharin in Schwartz et al. (2017). The left panel shows the percentages of choices for heroin in individual subjects when the FR for both heroin and saccharin was 1. The right panel shows percent heroin choice when the FR for saccharin was increased over sessions whereas the FR for heroin remained at 1. Reprinted from Schwartz et al. (2017) with permission from Elsevier

In some studies, the price of the option chosen most frequently has been increased to test whether preference shifts to the initially nonpreferred option. For example, Schwartz et al. (2017) first trained rats to choose between heroin (0.02 mg/kg/infusion) and saccharin on a discrete-trials procedure like that described above. When both reinforcers were available on a FR 1 schedule, rats chose saccharin on most trials (Fig. 2a), but as the FR for saccharin was increased, rats took increasingly more heroin infusions, until at FR 32 for saccharin, they took heroin on approximately 70% of trials (Fig. 2b). Similar results have been found in rats choosing between cocaine and saccharin when the price of saccharin was increased and the price of cocaine held low (Cantin et al., 2010; Kim et al., 2018). These results show that increasing the price of a reinforcer causes a decrease in consumption of that reinforcer, consistent with the law of demand.

A fall in the level of consumption of the reinforcer that has increased in price below that of the initially preferred reinforcer has often been interpreted as a reversal of preference. This makes sense if the relative allocation of choices is taken as the measure of preference. But if the allocation of lever presses, rather than choices, reflects preference, then a very different picture emerges. For example, in the Schwartz et al. experiment described above, when the prices of heroin and saccharin were both FR 1, rats allocated approximately 70% of the total lever presses that they made to saccharin. But when the price of saccharin was raised to FR 32, rats made over 90% of their total lever presses on the saccharin lever. Thus, rats increased their relative responding for saccharin as it became more expensive. Similar results have been observed with other drug and nondrug reinforcers. For example, Marchant et al. (2023) found that rats choosing between alcohol and social interaction, when both were available on an FR-1 schedule, made about 80% of their choices for alcohol. When the price of alcohol was raised to FR 24 (and social interaction kept at FR 1), rats obtained approximately equal numbers of alcohol and social reinforcers, which was interpreted as a decrease in preference for alcohol. But rats actually increased the percentage of lever presses made for alcohol to approximately 95% in order to obtain equal numbers of alcohol and social reinforcers.

**Fig. 3:**
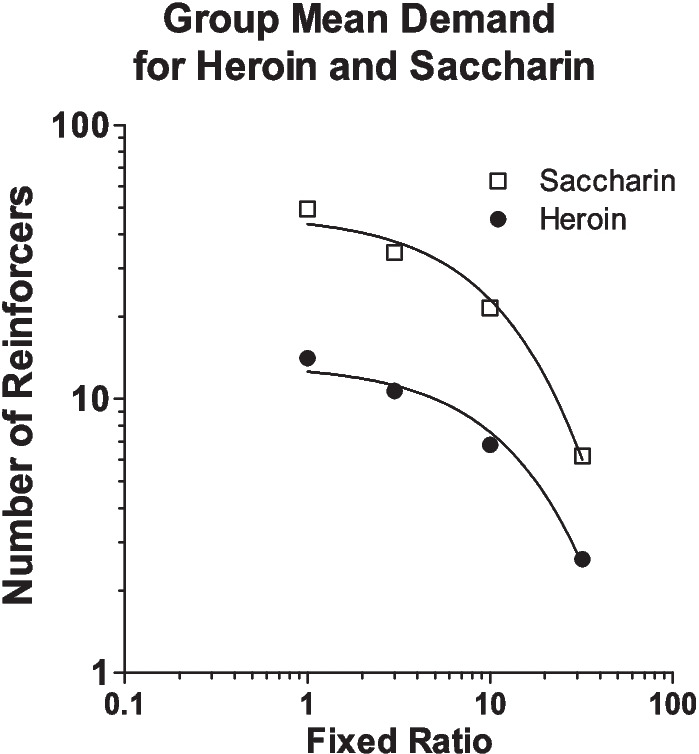
Demand curves for saccharin and heroin from the Schwartz et al. (2017) study. Rats worked for either saccharin or heroin with only one of these reinforcers available at a time (i.e., they were not choosing between them). Reprinted from Schwartz et al. (2017) with permission from Elsevier

Should number of reinforcers obtained or relative responding across levers be taken as the indication of rats’ preference? A view introduced by Bickel and Madden (1999) suggests an alternative way of thinking about choice and preference. For each reinforcer, there is an underlying demand curve. In a choice situation, the number of each type of reinforcer obtained depends on which point on each reinforcer’s demand curve corresponds to the prices at which the reinforcers are offered on the choice procedure. For example, in the Schwartz et al. study, demand curves were generated for both heroin and saccharin separately before allowing rats to choose between them. These are shown in Fig. 3. When each reinforcer was available on its own at FR 1, rats took about 14 heroin infusions and 50 saccharin reinforcers, meaning that heroin represented about 22% of the total reinforcers obtained at FR 1. This is not too different from the approximately 30% of heroin reinforcers obtained during choice when both heroin and saccharin were available at FR 1. Looking at the demand curves in Fig. 3, where only one reinforcer was available at a time, rats took 14 heroin infusions when the heroin price was FR 1 and they took 6 saccharin reinforcers when the saccharin price was 32. Extrapolating from these single-reinforcer demand curves, it would be expected that rats would take more heroin infusions than saccharin reinforcers during choice when the price of heroin was FR 1 and the price of saccharin was FR 32. To be more precise, it may even be expected that, at these prices, rats would choose heroin and saccharin reinforcers in an approximately 14:6 ratio, which corresponds closely to the approximately 70% of choices that rats made for heroin (see Fig. 2b). Bickel and Madden’s approach can also explain why the relative allocation of lever presses to saccharin increased as its price increased. As noted when demand curves were introduced, price increases cause the number of responses made for a reinforcer to increase (until P_max_ is reached). When lever pressing for saccharin alone (see demand curve), rats in the Schwartz et al. study obtained about 50 saccharin reinforcers at FR 1 and 6 reinforcers at FR 32, meaning rats increased their response output on the saccharin lever from 50 to approximately 200 lever presses across these prices. In the choice situation, as the price of saccharin was increased from 1 to 32 lever presses, rats similarly increased their response output on the saccharin lever.

Overall, the results of the Schwartz et al. (2017) experiment support Bickel and Madden’s (1999) point that choice between two reinforcers can be predicted from their underlying single-reinforcer demand curves. This suggests that when we ask which of two reinforcers a rat prefers, we may really be asking “at which point on each reinforcer’s demand curve will consumption of reinforcer X (or allocation of responses to X) be higher than that of reinforcer Y?” If rats reproduce in a choice situation what they what would have done if only one reinforcer were available, this may raise the question of whether there is anything special about choice behavior above and beyond the joint observation of two points from separate demand curves. As will be seen later, there are situations where it would be difficult to predict choice behavior solely on the basis of the individual demand curves for each reinforcer. This is especially evident when the two reinforcers economically interact, a behavioral economic concept to be addressed next.

## Economic Interactions between Drug and Nondrug Reinforcers

When the two reinforcers economically interact, price changes can have opposite effects depending on whether the reinforcers interact as substitutes or complements. These labels are not discrete categories but rather describe the ways in which two reinforcers interact along a continuum, with perfect substitutes at one end, perfect complements at the other, and independents in the middle (Allison, 1983; Green & Freed, 1993). How the allocation of behavior changes when the relative prices of the two reinforcers are changed tells us how two reinforcers interact along the continuum (Green & Rachlin, 1991).

Substitutes generally serve a similar function. For example, oranges and tangerines both provide a sweet, citrusy taste, vitamin C, etc. If reinforcer X and Y are substitutes, increases in the price of X relative to Y causes increased allocation of behavior (or spending) to Y (with a consequent increase in consumption of Y and decrease in consumption of X). If a person who buys some oranges and some tangerines each month found one day that the price of oranges has doubled while the price of tangerines has halved, they would likely increase their relative allocation of money to tangerines. Independents are functionally unrelated (e.g., toothpaste and socks). Changes in the relative prices of independents do not produce changes in the relative allocation of behavior (assuming those price changes do not affect subjects’ income too much).

Complements are perhaps the most interesting case. Complements are typically consumed in a relatively fixed proportion (e.g., one cup of cooked pasta and half a cup of pasta sauce). If X and Y are complements, subjects prefer a smaller bundle containing both X and Y in a particular ratio over larger bundles containing exclusively one or the other. In the extreme, one member of a pair of complements may be close to worthless without the other (e.g., a jar of pasta sauce with no pasta to put it on). In a less extreme case, reinforcer X may increase the value of reinforcer Y even if reinforcer Y has some value alone (e.g., tonic makes gin more palatable). With complements, increases in the relative price of X cause an increase in the relative allocation of behavior to X, an outcome Green and Rachlin (1991) referred to as “anti-matching.” This is somewhat counterintuitive—why increase spending on the option that has become more expensive? The reason is that allocation of behavior to the now more expensive option must increase if the subject is to maintain consumption of the two at the preferred ratio. For example, if the price of pasta sauce halved and the price of pasta doubled, the person would have to increase their relative allocation of money to pasta in order to maintain consumption of the two at the same ratio as previously.

Studies investigating choice between drug and nondrug alternatives in rats have begun to explore these reinforcer interaction dynamics (Beasley et al., 2024; Bird et al., 2024; Bunney et al., 2023; Smith et al., 2023). In a recent experiment, Beasley et al. (2024) investigated how heroin or cocaine interacted with the nondrug alternatives: timeout-from-avoidance (TOA) or saccharin. TOA, a period of signaled safety from shock, is a negative reinforcer that can maintain high rates of responding on ratio schedules (Galizio & Allen, 1991). This makes it possible to manipulate its price in the same way that the price of drugs or saccharin can be manipulated. The income-compensated price-change method (for full description and rationale, see Allison, 1983; Green & Freed, 1993; Green & Rachlin, 1991) was used to assess substitute/complement interactions. Rats were given a budget of responses (e.g., 360 lever presses) that they could choose to spend on the drug reinforcer or the nondrug alternative. Once rats spent their budget, the session ended. At first, the drug and nondrug reinforcers were available at an equal price (FR 12). Then the relative prices of the two reinforcers were varied to determine how the allocation of behavior shifted. The budget of responses available to the rat was adjusted with each price change to control for possible income effects (described below). There was no ITI between choices (as in the discrete-trials procedure) to allow for potential reinforcer interactions to be revealed. In studies using the income-compensated price change method (e.g., Freed & Green, 1998; Green & Rachlin, 1991; van Wingerden et al., 2015), it is common for relative price to be manipulated by reducing the price of one reinforcer and simultaneously increasing the price of the other, as was done here. In such studies, relative price is the key independent variable. But it should be noted that when the prices of both reinforcers are simultaneously changed, it is unknown whether the decrease in the price of one reinforcer was more important than the increase in price of the other for the changes in behavior observed.

**Fig. 4:**
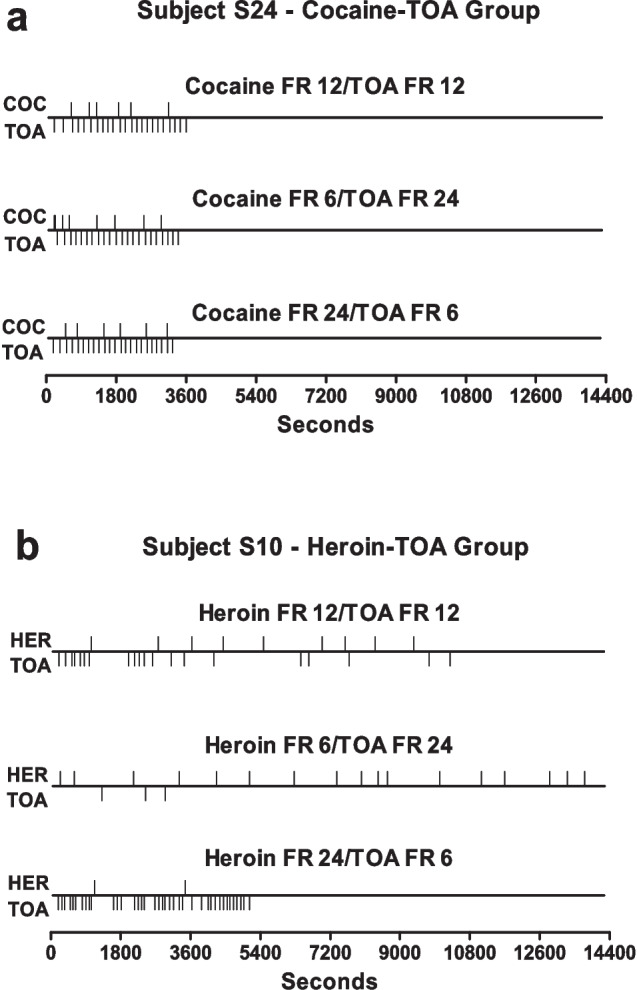
Panel (**a**) presents event records from a representative subject choosing between cocaine and timeout-from-avoidance (TOA) across the various price combinations tested in the Beasley et al. (2024) study. Upward ticks represent drug infusions and downward ticks represent TOA reinforcers. Panel (**b**) presents event records from a representative rat choosing between heroin and TOA. Reprinted from Beasley et al. (2024) with permission from the American Psychological Association

Beasley et al. (2024) found that cocaine (0.5–0.75 mg/kg/infusion, adjusted for individual rats) and TOA were complements in rats. Event records from a representative rat illustrate this interaction well (Fig. 4a). The key feature to notice is that this rat obtains virtually the same proportions of cocaine and TOA reinforcers, and in roughly the same distribution over time, across conditions despite a four-fold shift in the reinforcers’ relative prices. The rat could only accomplish this constancy of intake of the two reinforcers across the changing prices by increasing its allocation of behavior to the option that became more expensive—i.e., by “anti-matching” (Green & Rachlin, 1991). This pattern tells us that the rat prefers a smaller bundle of both cocaine and TOA, rather than a larger bundle containing more of just one or the other. This outcome suggests that cocaine may increase the reinforcing value of TOA, or vice-versa, in such a way that the two are most valuable when consumed together.

Heroin (0.02–0.03 mg/kg/infusion, adjusted for individual rats) and TOA, on the other hand, were partial substitutes in rats. (This was found in a separate group of rats that chose between heroin and TOA, rather than between cocaine and TOA, as in the group described in the previous paragraph.) Subjects choosing between heroin and TOA increased their relative allocation of responses to the option that became cheaper upon price changes. Figure 4b shows event records from a representative rat. In contrast to the rat choosing between cocaine and TOA, this rat obtained many more heroin reinforcers when it was cheap and many more TOA reinforcers when it was cheap. The finding of substitutability suggests that heroin and TOA serve a similar function in rats. Beasley et al. proposed that the analgesic or anxiolytic effect of heroin may have provided relief from the aversiveness of the avoidance situation and this relief was functionally similar to the relief provided by the cued period of safety from shock. In a second experiment, Beasley et al. found that heroin (0.03 mg/kg/infusion) and saccharin were complements, whereas cocaine (0.3–0.75 mg/kg/infusion, adjusted for individual rats) and saccharin were independents.

Smith et al. (2023) recently found that cocaine (0.5 mg/kg/infusion) and social reinforcement were substitutes in rats. They used a behavioral economic approach different from the income-compensated price-change method described above. Rats worked for either cocaine or social reinforcement by pressing one lever across prices varying from FR 1 to FR 15. They could press a second lever for the other reinforcer, which was always available at a constant price of FR 1. Cross-price elasticities of demand (reflecting the percent change in consumption of the constant-price reinforcer as a function of the percent change in the price of the varying-price alternative) were between approximately 0.2 and 0.4 for cocaine and social interaction with a cocaine-free partner, respectively, indicating these goods were partial substitutes. Smith et al. also investigated the impact of the partner being under the influence of cocaine and found that this made cocaine no longer substitute for the partner, whereas the partner (whether under the influence of cocaine or not) continued to substitute for cocaine. In a recent study using the income-compensated price-change method, Bird et al. (2024) also found evidence that cocaine (0.125–0.75 mg/kg/infusion, adjusted for individual rats) and social reinforcement were substitutes, whereas heroin (0.03 mg/kg/infusion) and social reinforcement were economic independents.

To summarize the experiments with cocaine and heroin described above, each drug was found to be either a substitute, independent, or complement, depending on the specific nondrug reinforcer used. Cocaine was a substitute for social interaction (Bird et al., 2024; Smith et al., 2023), independent with respect to saccharin (Beasley et al., 2024), and a complement to TOA (Beasley et al., 2024). Heroin, on the other hand, was a substitute for TOA (Beasley et al., 2024), an independent with respect to social interaction (Bird et al., 2024), and a complement to saccharin. In a recent study using nicotine as the drug reinforcer in rats, Bunney et al. (2023) found that sucrose substituted for nicotine, but not vice-versa.

The pattern of relationships that a drug has with different nondrug reinforcers could provide new insights into their profile of effects in rats. For example, substitution of cocaine for social interaction suggests that these two reinforcers serve a similar function. The common element could be stimulation or arousal, provided by the partner rat in the case of social interaction and provided by pharmacological effects (e.g., sympathetic nervous system activation) in the case of cocaine. The finding that heroin and social interaction were independents suggests that, in contrast, heroin does not have arousing effects similar to those produced by social interaction. Instead, the finding that heroin substituted for TOA suggests quite the opposite—that it may be reinforcing, in part, due to calming effects that may be similar to those experienced during a reprieve from a stressful situation. By further exploring these relationships, and how they might change with repeated drug use, this behavioral economic approach could provide new information on motives for drug use in different situations.

## Economy Type and Choice between Drug and Nondrug Reinforcers

Economy type is another economic variable that can affect choice behavior (Hursh, 1978, 1980). In a closed economy, the experimental session is the only opportunity to obtain the reinforcer. For example, if the only source of food is that obtained for lever pressing in the operant chamber, the economy for food is closed. In an open economy, however, there is more than one source of the reinforcer. If a rat earns food for lever pressing during the experimental session but also receives postsession supplemental food provided by the experimenter, or has some other opportunity to obtain food, the food economy is open. As Hursh (1980, 1984) explained, the feedback function between behavior and the outcome differs across closed and open economy types. In a closed economy, receipt of the reinforcer is wholly dependent on behavior. In an open economy, there is some degree of independence between behavior and outcome.

From a behavioral economic perspective, opening the economy for a reinforcer should decrease the likelihood that it will be chosen, as compared to when it is available in a closed economy. If the subject has limited opportunity to choose between two reinforcers and the economy for one is open while the economy for the other is closed, the rational response is to spend those limited choices on the closed-economy reinforcer. Any open-economy reinforcers forgone during choice can be replaced outside of the choice situation. Whether this effect of opening the economy is observed will depend on the ability of temporally delayed reinforcers to substitute for immediate reinforcers of the same kind, and potentially other variables.

**Fig. 5:**
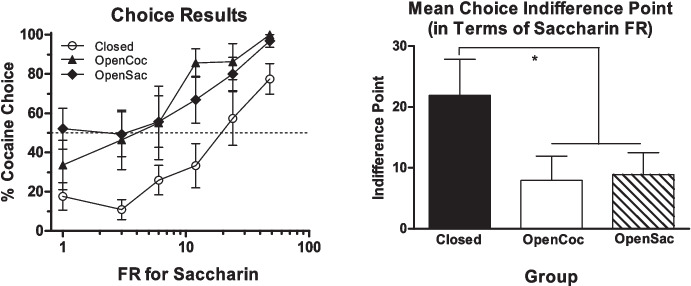
The left panel shows the percentage of trials on which cocaine was chosen (over saccharin) in the closed economies group (“Closed”), open cocaine economy group (“OpenCoc”), and open saccharin economy group (“OpenSacch”) in the Kim et al. (2018) study when the price of saccharin was increased over sessions (the price of cocaine was always FR 1). The right panel shows the group mean indifference points (the estimated price at which percent cocaine choice was 50%). Reprinted from Kim et al. (2018) with permission from Elsevier

**Fig. 6:**
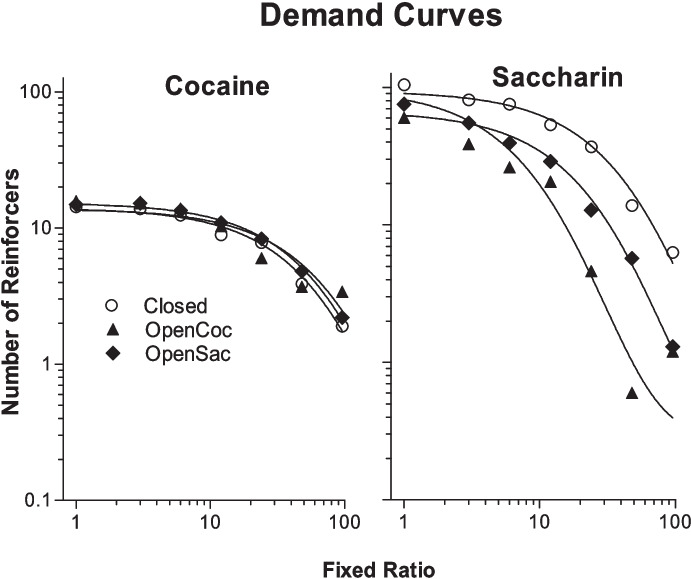
Demand curves for cocaine (left panel) and saccharin (right panel) in the three groups from the Kim et al. (2018) study. Only one reinforcer was available at a time during demand testing. Reprinted from Kim et al. (2018) with permission from Elsevier

Kim et al. (2018) tested this prediction in rats choosing between cocaine (0.75 mg/kg/infusion) and saccharin. Three groups of rats were trained on a discrete-trials choice procedure. For one group, the choice session was the only opportunity for rats to obtain either cocaine or saccharin. The economy for both reinforcers was closed for this group. For a second group, the cocaine economy was open. They had access to cocaine on an FR 1 schedule for a 3-h period following each choice session. For a third group, the economy for saccharin was made open by providing postsession access to saccharin. Figure 5 shows the results. Opening the saccharin economy indeed made rats less likely to choose saccharin during the session and therefore promoted choice of cocaine. It is surprising, however, opening the cocaine economy did not reduce choice of cocaine. Instead, rats with postsession cocaine access were more likely to choose cocaine than the group for which the economies for cocaine and saccharin were both closed. In the open cocaine economy group, the increased preference for cocaine over saccharin was thought to be due to the formation of a conditioned-taste-aversion-related association between saccharin and postsession cocaine. This conclusion was based on the observation that when demand for cocaine and for saccharin was assessed with only one of these reinforcers available at a time, the elasticity of demand for cocaine was unaffected by opening the cocaine economy or the saccharin economy (Fig. 6, left panel), but demand for saccharin became more elastic both when the saccharin economy was opened and when the cocaine economy was opened (Fig. 6, right panel).

A subsequent experiment (Gunawan et al., 2020) with rats choosing between heroin (0.03 mg/kg/infusion) and saccharin found that opening the saccharin economy increased allocation of choices to heroin, whereas opening the heroin economy had no effect. In a related study, a separate experiment assessing demand for heroin (0.03 mg/kg/infusion) or saccharin when either was available on its own, found that opening the heroin economy had no impact on demand for heroin, but opening the saccharin economy weakened demand for saccharin (Gunawan et al., 2019). It is unclear why opening the saccharin economy had the expected effect, but opening the heroin or cocaine economy did not. This surprising outcome will be discussed in a later section on instances where rats’ drug versus nondrug reinforcer choice behavior seemingly does not conform with behavioral economic principles. But first, the final concept—income effects—will be discussed.

## Income Effects and Choice between Drug and Nondrug Reinforcers

Income effects can also affect the allocation of behavior between alternatives (Allison, 1983; Hursh, 1984). The concept of elasticity of demand, introduced previously in relation to the effect of price changes, also applies to changes in income. For reinforcers for which income-elasticity of demand is high, reductions in income produce relatively large decreases in consumption. Such reinforcers are typically thought of as luxuries. For example, if a person’s weekly work hours are cut and therefore income is reduced, spending on luxuries like sports and concert tickets, restaurant meals, etc., is often cut back first, or even eliminated altogether. For reinforcers with low income-elasticity of demand, decreases in income produce relatively small decreases in consumption. These kinds of goods are often described as necessities. For example, even when income is reduced, the person will continue to pay for rent, buy supermarket food (perhaps somewhat less than previously), etc. By reducing spending on luxuries to a greater degree than that spent on necessities, the proportional allocation of spending to necessities has increased.

In experiments with animals, income changes are modelled by varying the number of responses or the number of choices the subject can make per session. When income is high, the animals can afford to take as many of both reinforcers as it likes; choices of one reinforcer have little to no impact on consumption of the other. But when the animal can only make a few choices per session (i.e., when income is low), choice of one will necessarily reduce consumption of the other. This constraint may be especially relevant to interpreting the results of experiments using discrete-trials choice procedures with long ITIs and few choice opportunities per session. A procedure where the subject can make, for example, 15 choices between heroin and social reinforcement, as in the Venniro et al., (2018, 2019) studies, may represent a very low-income condition. Now, it is not the case that choice of one reinforcer has little impact on consumption of the other; instead, there are relatively large opportunity costs associated with each choice. If the difference between reinforcers in income-elasticity of demand is great enough, one reinforcer may not be chosen at all in very low-income situations. This could potentially explain why, if heroin and social reinforcement are independents (Bird et al., 2024), rats voluntarily abstained from heroin in the Venniro et al. experiments when rats were offered social interaction as an alternative.

A study using baboons as subjects illustrates how the effect of income on drug versus nondrug reinforcer choice can be investigated in animal models. In one of the earliest applications of behavioral economics to an animal model of drug taking, Elsmore et al. (1980) allowed baboons to choose between heroin and food in discrete trials that occurred throughout the day. The economies for food and heroin were both closed. Income, or the number of choices that subjects could make per day, was manipulated by varying the ITI between choices. In an initial condition, the ITI was 2 min, which allowed subjects to make many choices per day. In this high-income condition, baboons took roughly equal numbers of food and heroin reinforcers, roughly 80 of each. Then, the ITI was increased over phases up to 12 min, which permitted relatively few choices per day. As income was reduced, there was a large decrease in choices of heroin, but only a small reduction in choices for food. As a result, as income decreased, baboons greatly reduced the percentage of trials on which they chose heroin over food. In other words, demand for heroin was relatively income elastic, whereas that for food was income inelastic.

Gunawan et al. (2020) applied the approach to modeling income effects used by Elsmore et al. to rats’ choice between heroin (0.03 mg/kg/infusion) and saccharin. A key part of the experiment was testing the hypothesis that income effects and economy type effects interact. In a closed economy, a reduction in income should promote increased allocation of choices to the reinforcer with lower income-elasticity of demand (i.e., the necessity-like good). But if the economy for that reinforcer were opened whereas that of the other reinforcer were closed, this income effect should be weakened, or even reversed. The rationale is that if animals could obtain a reinforcer freely outside of the choice session, they would be less inclined to spend their limited income on that reinforcer, even if demand for it were income inelastic, and instead choose the other reinforcer to which they have no outside access.

To test this hypothesis, Gunawan et al. (2020) trained three groups of rats on a discrete-trials choice procedure where heroin and saccharin were the reinforcers. Income was varied over phases in each group by changing the ITI, and therefore changing the number of choices rats could make per session. In the high-income, moderate-income, and low-income conditions, the ITIs were 20 s, 180 s, and 600 s, respectively, allowing for 540, 60, and 18 choices over the 3-h session. For the closed economy group, the session was rats’ only opportunity to obtain heroin or saccharin. For the heroin open economy group and the saccharin open economy group, heroin or saccharin, respectively, was available for 3 h on an FR-1 schedule following each choice session.

**Fig. 7:**
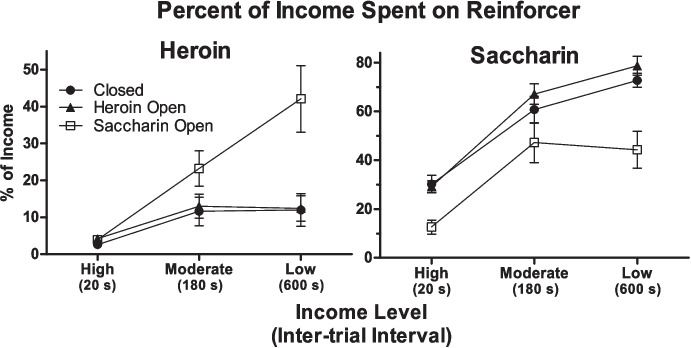
Percent of income (choices) spent on heroin (left panel) or saccharin (right panel) across income conditions in the closed economies group (“Closed”), open heroin economy group (“Heroin Open”), and open saccharin economy group (“Saccharin Open”) from the Gunawan et al. (2020) study. Reprinted from Gunawan et al. (2020) with permission from Springer

The main result is presented in Fig. 7. In the closed economy group and the open heroin economy groups, the allocation of income (i.e., choices) to heroin was low across income conditions (left panel). (Note that % income here is the percentage of choices for heroin or saccharin out of the maximum possible choices that rats could have made. The percentages for the two reinforcers do not sum to 100% due to omissions.) Allocation of income to saccharin (right panel), on the other hand, rose from approximately 30% to about 70% as income was reduced in these groups. For the open saccharin economy group, in contrast, the reduction in income caused an increase in the percentage of income that rats allocated to heroin from approximately 4% to 40%. Furthermore, the percentage of income allocated to saccharin was reduced compared to the other two groups across income levels. The results of Gunawan et al. (2020) show that income effects can alter rats’ choice between heroin and saccharin. They also provide evidence in support of the hypothesis that the effect of income depends on economy type.

A unifying principle that may help to explain both income effects and economy type effects is contingency. In a drug versus nondrug choice situation, multiple contingencies potentially operate simultaneously depending on the specific procedures used. Consider the case when the discrete-trials mutually exclusive choice procedure with long ITIs is used and the choice session is the only opportunity to obtain either reinforcer. In behavioral economic terms, income is low and the economies for both reinforcers are closed. There are (at least) four contingencies operating: (1) a response on the drug lever results in drug; (2) a response on the drug lever results in reduced consumption of the nondrug alternative; (3) a response on the nondrug alternative lever results in delivery of the nondrug alternative; and (4) a response on the nondrug alternative lever results in reduced consumption of the drug.

Now suppose income is increased by eliminating the ITI and allowing rats to freely choose between the two reinforcers as often as they like for the duration of the session. In this situation, contingencies #2 and #4 are essentially removed—there is no longer an opportunity cost in terms of reduced consumption of one reinforcer when the other is chosen. The increase in income should, in theory, result in increased consumption of both reinforcers, but a proportionally larger increase in consumption of the reinforcer that was previously associated with the greater opportunity cost. Economy type manipulations also modify the contingencies described above. For example, for a rat trained on the original discrete-trials mutually exclusive choice procedure described above, opening the economy for the nondrug reinforcer by providing post-choice-session access weakens contingency #2 from above. Now choosing the drug no longer reduces daily consumption of the nondrug alternative because the subject can obtain it freely outside of the choice session.

This analysis of economy type and income effects suggests that changes in the broader behavioral economic context can affect the contingencies in effect at the time of choice. Whether this affects choice behavior depends on the subject being able to integrate, at some level, the consequences of their immediate behavior with what occurs outside of the choice situation. In some cases, rats appear to be able to do this; in others, however, local and immediate factors may be the strongest or even the sole determinant of behavior, as will be discussed in the next section.

## When Explanations Other than Behavioral Economics are Needed

The review so far has described results from drug versus nondrug choice studies in rats that were largely consistent with the predictions of behavioral economics. This section will discuss instances where additional, or alternative, explanations of behavior are necessary.

### Lack of Effect of Opening the Drug Economy

The first of these was briefly described in the section on economy type effects. Opening the economy for cocaine or heroin, by providing post-choice-session access to these drugs, did not reduce rats’ choice of the drug over saccharin as expected (Gunawan et al., 2020; Kim et al., 2018). Likewise, postsession access to these drugs had no effect on demand for these drugs (Gunawan et al., 2019; Kim et al., 2018). This was surprising because opening the saccharin economy in the same way reduced choice of saccharin as well as demand for saccharin, as expected from a behavioral economic perspective.

A similar lack of an effect of opening the drug economy has been found in experiments using monkeys, rather than rats, as subjects. For example, Banks and Negus (2010) allowed monkeys to choose between cocaine and food across two conditions. In one condition, which can be described as a closed cocaine economy, the choice session was monkeys’ only opportunity for cocaine. In a second condition, which can be described as an open cocaine economy, each choice session was followed by a 21-h supplemental session where monkeys could self-administer cocaine on its own at a relatively low price. Banks and Negus (2010) found that post-choice-session supplemental cocaine had no impact on the relative allocation of responses to cocaine or food. Negus (2006) found similar results in monkeys choosing between heroin and food. The lack of an effect of opening the drug economy on choice in monkeys contrasts with the effect of opening the food economy. Nader and Woolverton (1992) trained monkeys to choose between food and cocaine. When the food economy was opened by providing post-choice-session food, choice of cocaine increased, as would be expected by behavioral economics.

Differences in delay discounting processes between drug and nondrug reinforcers might be thought to potentially explain these outcomes. Previous studies have shown that the timing of postsession supplemental food can affect responding for food during the session. For example, Smethells et al. (2012) found that providing immediate postsession supplemental food reduced progressive-ratio breakpoints for food compared to when the postsession supplemental food was delayed several hours. Similar results suggesting that the impact of postsession food is discounted by delays were found by Bacotti (1976) and Timberlake et al. (1987). The lack of effect of opening the drug economy on animals’ responding for the drug, together with the expected effect of opening the food or saccharin economy, could potentially be explained if animals discount postsession drugs to a greater extent than they discount postsession nondrug alternatives. However, research on discounting has found that, if anything, rats and monkeys discount cocaine to a *lesser* degree than they discount delayed food (Huskinson et al., 2015, 2016; Smethells et al., 2016).

Differences between drug and nondrug alternatives in the way that current and future reinforcers are integrated could potentially explain the differing effects of opening the drug versus nondrug economy in animal studies. Timberlake et al. (1987) note that the time horizons over which reinforcers are integrated may differ for different behavior systems. It may be that feeding and drinking systems evolved such that future availability/nonavailability of food or fluids importantly determines current behavior. If, for example, an energy deficit now can be tolerated without significant negative consequences, it may be advantageous to reduce the time and effort spent on foraging now if food will be abundant later and energy stores can be repleted. However, take for example, a reinforcer like warmth in cold rats (Weiss & Laties, 1960). It seems unlikely that a cold rat would reduce its responding for warmth now due to the later availability of abundant warmth. In a similar way, when it comes to i.v. drug self-administration, the current state of the animal may be more important than potential future availability of drugs.

### Species Differences

It is interesting that in contrast to what was observed in rats and monkeys, humans’ drug choice behavior does appear to be affected by opening the drug economy in the predicted way. For example, Mitchell et al., (1994, 1998) demonstrated this outcome in a study with humans choosing between money and cigarettes. Making cigarettes freely available after the choice session decreased allocation of choices to cigarettes during the session. Mitchell et al. (1995) found that opening the coffee economy had the expected effect in humans choosing between coffee and money as well. Greenwald and Steinmiller (2009) found a similar effect of opening the hydromorphone (Dilaudid) economy in humans choosing between this opioid and money. Informing subjects that free hydromorphone would be available after the session reduced their choice of hydromorphone over money.

One difference between animal and human studies is that money is typically used as the nondrug alternative reinforcer in human studies. Money is a special type of reinforcer in that it can be exchanged for many other commodities. In contrast, the nondrug alternatives used in animal studies, such as a food pellet, are not fungible in the way that money is for humans. Might the fungibility of money account for the differences in the impact of opening the drug economy across the animal and human studies described above? Considering, for example, the Greenwald and Steinmiller (2009) study, it seems unlikely that the effect of opening the hydromorphone economy—decreased choice of hydromorphone during the choice session—would not have occurred if a less fungible nondrug alternative (e.g., a candy bar) were used instead of money. If anything, a case could be made that use of money instead of a less fungible nondrug alternative could have made it harder to find an effect of opening the hydromorphone economy. If it were relatively easy to exchange money for extra-session opioids but difficult to exchange a less fungible nondrug alternative for extra-session opioids, then choice of money might be expected to be less sensitive to the availability of postsession hydromorphone (because the money could in theory be used to buy other opioids outside of the session) than would be the choice of a less fungible nondrug alterative.

One potentially important difference between the human and animal studies is that humans are instructed of postsession drug availability. Humans can use this information to reason through a choice strategy that maximizes benefits and minimizes costs. Rats and monkeys, on the other hand, must learn through repeated experience of the availability of postsession drug. It could be that the ability to reason and plan in a way that incorporates future reinforcer availability is why an effect of opening the drug economy is seen in humans, but not animals. But this still does not explain why in animals an effect of opening the economy for nondrug reinforcers is seen. Future research, drawing on concepts from outside of behavioral economics, will likely be needed to answer this question.

### Impact of Antecedent Stimuli on Choice between Drug and Nondrug Reinforcers

Recent research by Vandaele et al., (2019, 2020, 2022) illustrates another situation where behavioral economic explanations do not account well for rats’ drug versus nondrug reinforcer choice behavior. They showed that rats’ choices between cocaine (~ 0.75 mg/kg/infusion) and nondrug reinforcers can become habitual, where habitual responding is defined in terms of insensitivity to changes in outcome value (Balleine & Dickinson, 1998; Corbit, 2018; Thrailkill et al., 2018). In particular, behavior is said to be habitual when that behavior is unaffected by manipulations that devalue the outcome; behavior is said to be goal-directed when it is affected by outcome devaluation. Vandaele and colleagues found that when rats are trained on a discrete-trials procedure to choose between cocaine and saccharin (Vandaele et al., 2020, 2022) or water (Vandaele et al., 2019), choice of the nondrug alternative can become resistant to manipulations that devalue it. For example, when rats were given free access to water both prior to the session and during the session, they still chose water over saccharin, even though they often did not drink the water when chosen (Vandaele et al., 2019). They also found that after rats learned to choose between cocaine and saccharin, devaluing saccharin, by either providing rats free saccharin to the point of satiation or by pairing saccharin with lithium chloride, did not make rats stop choosing saccharin over cocaine (Vandaele et al., 2020, 2022).

From a behavioral economic perspective, these are surprising results. Devaluing the nondrug alternative to the degree that it is no longer consumed, and presumably has little to no value, should have increased choice of cocaine. As Vandaele et al. note, rats would take cocaine when it was the only option offered; it was only when cocaine was offered as a concurrently available alternative to water or saccharin that rats seemingly refused it. To use Vandaele et al.’s expression, it appeared that cocaine fell into oblivion when presented in the choice situation. They proposed that the formation of habitual water and saccharin choice they observed could have been due to features of the discrete-trials procedure commonly used in rat choice studies. In a previous study, Vandaele et al. (2017) found that habitual responding for food developed when rats were trained on a procedure where a lever was intermittently presented to rats and followed by response-contingent food, whereas the behavior of rats trained to press a continually present lever remained goal-directed. It could be that the intermittent presentation of levers on the discrete-trials choice procedure promotes habitual responding in a similar way.

Behavioral economics, rooted as it is in operant theory, places much focus on the consequences of a behavior. Habitual behavior, on the other hand, is driven by antecedents, rather than consequences. In particular, the stimulus–response (S-R) associations that are learned between cues (e.g., the saccharin lever) and the response (e.g., lever pressing) are thought to control behavior rather than the response-outcome (R-O) operant contingency, which is thought responsible for goal-directed behavior (Corbit, 2018). The results of the Vandaele et al. studies show that antecedents can sometimes be even more important than consequences in drug versus nondrug choice behavior. In general, these results remind us that drug versus nondrug choice procedures in rats are fundamentally learning tasks—and of the kind that can allow for various forms of learning to be expressed. Not only do rats learn the operant contingencies that are often the focus of behavioral economics, but, depending on the procedure, they can also learn S-R associations like those described above as well as Pavlovian stimulus–stimulus (S–S) associations that can be expressed, for example, in the form of Pavlovian sign-tracking (e.g., see Madsen & Ahmed, 2015). Choice is complex even when only considering it in terms of operant contingencies. Adding the influence of other forms of learned behavior on top of this increases the complexity even further.

## Conclusion

To conclude, it is hoped that this review shows how a behavioral economic perspective can contribute to understanding the results of drug versus nondrug alternative choice experiments in rats. By considering price effects, substitute/complement interactions, economy type, and income effects we can better appreciate the various potential factors that determine rats’ allocation of behavior between drug and nondrug alternatives. Keeping such factors in mind can help in comparing results from choice studies using different procedures (e.g., discrete-trials versus continuous availability of choice options, different combinations of drug and nondrug options). It will be important to also appreciate the sometimes critical role played by learning factors unrelated to behavioral economics in determining rats’ choice behavior. By further refining our thinking about animal models of addiction, we can come closer to accurately capturing the processes that contribute to drug use in humans.

## Data Availability

Because this is a review article, no new data are presented.
